# A prospective longitudinal multicentre study of health related quality of life in ICU survivors with COPD

**DOI:** 10.1186/cc13019

**Published:** 2013-09-24

**Authors:** Johan Berkius, Lars Engerström, Lotti Orwelius, Peter Nordlund, Folke Sjöberg, Mats Fredrikson, Sten M Walther

**Affiliations:** 1 Department of Anaesthesia and Intensive Care, Västervik County Hospital, Östra Kyrkogatan 48, SE-593 81 Västervik, Sweden; 2 Division of Cardiovascular Medicine, Department of Medicine and Care, Faculty of Health Sciences, Linköping University, Garnisonsvägen 10, SE-581 85 Linköping, Sweden; 3 Department of Anaesthesia and Intensive Care, Vrinnevisjukhuset, Gamla Övägen 25, SE-601 82 Norrköping, Sweden; 4 Department of Intensive Care, Linköping University, County Council of Östergötland, Garnisonsvägen 10, SE-581 85 Linköping, Sweden; 5 Department of Clinical and Experimental Medicine, Faculty of Health Sciences, Linköping University, Garnisonsvägen 10, SE-583 85 Linköping, Sweden; 6 Department of Anaesthesia and Intensive Care, Ryhov Hospital, Husargatan 4, SE-551 85 Jönköping, Sweden; 7 The Burn Center, Department of Hand, Plastic Surgery and Intensive Care, County Council of Östergötland, Garnisonsvägen 10, SE-583 85 Linköping, Sweden; 8 Division of Occupational and Environmental Sciences, Department of Clinical and Experimental Sciences, Faculty of Health Sciences, Linköping University, Garnisonsvägen 10, SE-583 85 Linköping, Sweden; 9 Department of Cardiovascular Anaesthesia and Intensive Care, Linköping University Hospital, Garnisonsvägen 10, SE-583 85 Linköping, Sweden

## Abstract

**Introduction:**

Mortality amongst COPD patients treated on the ICU is high. Health-related quality of life (HRQL) after intensive care is a relevant concern for COPD patients, their families and providers of health care. Still, there are few HRQL studies after intensive care of this patient group. Our hypothesis was that HRQL of COPD patients treated on the ICU declines rapidly with time.

****Methods**:**

Fifty-one COPD patients (COPD-ICU group) with an ICU stay longer than 24 hours received a questionnaire at 6, 12 and 24 months after discharge from ICU. HRQL was measured using two generic instruments: the EuroQoL instrument (EQ-5D and EQ-VAS) and the Short Form 36 Health Survey (SF-36). The results were compared to HRQL of two reference groups from the general population; an age- and sex-adjusted reference population (Non-COPD reference) and a reference group with COPD (COPD reference).

**Results:**

HRQL of the COPD-ICU group at 6 months after discharge from ICU was lower compared to the COPD reference group: Median EQ-5D was 0.66 vs. 0.73, P = 0.08 and median EQ-VAS was 50 vs.55, P < 0.05. There were no significant differences in the SF-36 dimensions between the COPD-ICU and COPD-reference groups, although the difference in physical functioning (PF) approached statistical significance (P = 0.059). Patients in the COPD-ICU group who were lost to follow-up after 6 months had low HRQL scores at 6 months. Scores for patients who died were generally lower compared to patients who failed to respond to the questionnaire. The PF and social functioning (SF) scores in those who died were significantly lower compared to patients with a complete follow up. HRQL of patients in the COPD-ICU group that survived a complete 24 months follow up was low but stable with no statistically significant decline from 6 to 24 months after ICU discharge. Their HRQL at 24 months was not significantly different from HRQL in the COPD reference group.

**Conclusions:**

HRQL in COPD survivors after intensive care was low but did not decline from 6 to 24 months after discharge from ICU. Furthermore, HRQL at 24 months was similar to patients with COPD who had not received ICU treatment.

## Introduction

Survival after intensive care of patients with an acute exacerbation of chronic obstructive pulmonary disease (AECOPD) is poor. Almost 50% of patients die within two years of admission [1-3]. Important considerations for patients, their families and healthcare providers are not only survival but also health-related quality of life (HRQL) after intensive care [4]. There are only a few published reports on HRQL after intensive care in this group of patients [2,5]. In the COPD and Asthma Outcome Study (CAOS), HRQL was assessed in 291 AECOPD patients 6 months after ICU care in the United Kingdom using the EuroQoL 5-Dimension (EQ-5D) and 20-item Airways Questionnaire (AQ20) instruments [5]. These patients' HRQL scores were significantly lower than those in an age-matched general population, but similar to those of outpatients with COPD. Rivéra-Fernández and coworkers followed HRQL for six years in fifty patients with AECOPD at Spanish ICUs using a self-developed validated instrument [6]. They found a nonsignificant decline in HRQL scores in this selected cohort. These results from the United Kingdom and Spain were surprising to us, given the facts that COPD is a progressive disease and HRQL in COPD deteriorates in tandem with increasing disease severity and age [7]. Hence, the present study was undertaken to examine the development of HRQL from six months up to twenty-four months after intensive care. We hypothesized that HRQL declines with time in COPD patients after intensive care. We used the EQ-5D instrument and the Short Form 36 Health Survey (SF-36), both of which have been validated and are commonly used in evaluating the critically ill patient population [8-11]. The results were contrasted using measurements of HRQL in two reference groups, one with COPD and another without COPD, both derived from the general population in the same region as the studied ICUs.

## Materials and methods

In this prospective, longitudinal, multicentre study, we recruited patients between August 2000 and July 2004 at three mixed medical-surgical ICUs in southeastern Sweden. One is a university hospital and two are general hospitals. Each ICU admits 500 to 750 patients annually. Nearly all admissions to these three ICUs are emergencies, and the most common primary diagnoses are multiple trauma, sepsis, and disturbances in the respiratory or circulatory system or both. The Linköping University Regional Ethics Review Board approved the study protocol.

Patients ages 18 years and older who were admitted to and remained in the ICU for more than 24 hours (*N* = 1,663) were sent a letter inviting them to participate in a follow-up of their health. A set of structured questionnaires was mailed to survivors, who had given their consent to participate in the study, at six, twelve and twenty-four months after discharge from the ICU. Replies were initially received from 980 patients (59%). Their mean age was 58.2 years, and 42% were female. Selected results from this follow-up (LIVA Health-related quality of life after intensive care in adults Study) have been published previously [12].

**Figure 1:**
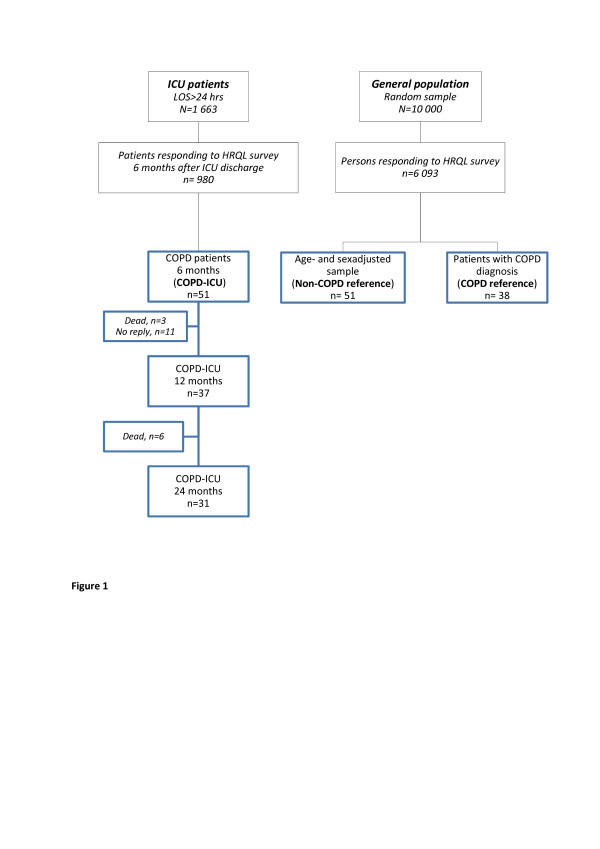
**Study outline**. COPD, chronic obstructive pulmonary disease; HRQL, health-related quality of life; LOS, length of stay.

We identified patients with COPD from among patients with respiratory disease in the LIVA Study (COPD-ICU group) by matching their data with information in the Swedish Hospital Discharge Register and the Cause of Death Register using the unique Swedish personal identity number. Patients with primary codes from the Tenth Revision of the International Classification of Diseases (ICD-10) that were compatible with COPD (J44.0, J44.1, J44.8 or J44.9) from hospital admission up to five years before study start were included in the COPD-ICU group. Patients who died within five years of the study start and had a primary or secondary cause of death compatible with COPD (ICD-10 codes cited above) on their death certificates were also included in the COPD-ICU group. Thus, 51 patients with COPD were identified using the criteria described above. Their HRQL scores were followed for 24 months after discharge from the ICU. Data for loss of responders are shown in the study flowchart in Figure 1. The clinical databases in each hospital were used to extract data on age, sex, reason for admission, Acute Physiology and Chronic Health Evaluation II (APACHE II) score, ICU length of stay (LOS) and time in hospital, time spent on ventilation and survival. We used the ICD-10 codes of prior hospital admissions found in the Swedish Hospital Discharge Register to compute the Charlson comorbidity index (CCI) score [13].

To contrast HRQL measurements in the COPD-ICU group, we used data from a public health survey performed in the same geographic region. The survey was mailed to 10,000 randomly selected individuals ages 20 to 74 years, from among whom 6,093 (61%) responded [14]. We formed a COPD reference group (*N* = 38) by matching the personal identity numbers of survey responders with information in the Swedish Hospital Discharge Register and the Cause of Death Register using the same ICD-10 codes as those used for the COPD-ICU group.

A non-COPD reference group (*N* = 51) was constructed by calculating age- and sex-adjusted HRQL scores. These HRQL scores were computed using coefficients that characterized linear relationships between individual HRQL domains, age and gender in the non-COPD responders of the public health survey, Health and Health Related Quality of Life as measured by the EQ-5D and the SF-36 in South East Sweden: Results from Two Population Surveys. Vital status was ascertained from the Cause of Death Register to analyze long-term survival.

### Assessment of health-related quality of life

The instruments chosen for the evaluation of HRQL were the EQ-5D and the SF-36 questionnaires [8,9]. Both are internationally accepted and have been recommended for measuring HRQL following critical care [10,15]. The EQ-5D involves a health state classification scheme of five items (mobility, self-care, usual activities, pain and/or discomfort and anxiety and/or depression), each having three alternatives [9]. Health states can be evaluated better than, equal to or worse than death. For health states evaluated as worse than death, the utility is negative [16]. The answers in the first part range from 0 to 1. The second part of the EQ-5D is a Visual Analogue Scale (EQ-VAS) ranging from 0 (worst possible health state) to 100 (best possible health state) on which the respondents rate how they perceive their health status.

The SF-36 instrument has 36 questions and generates a health profile based on eight subscale scores: physical functioning (PF), role limitations due to physical problems (RP), bodily pain (BP), general health (GH), vitality (VT), social functioning (SF), role limitations due to emotional problems (RE) and mental health (MH). The scores on all subscales are transformed to a single scale ranging from 0 (worst score) to 100 (best score) [17].

### Statistics

HRQL data are presented as median values and interquartile ranges because the measures are ordinal, bounded by lower and upper limits, and the number of observations is quite few. We also present mean values as this is typically done with HRQL data.

Student's *t*-test was used to compare groups at six months if the Shapiro-Wilk test indicated that distributions were normal; otherwise, the Wilcoxon rank-sum test was used. The Friedman test was used to examine repeated measurements in the same individual. A probability less than 0.05 was considered statistically significant. No adjustments were made for multiple testing in this study. The statistical analysis was performed using Stata/SE version 12 software (Stata Corp, College Station, TX, USA).

## Results

**Figure 2:**
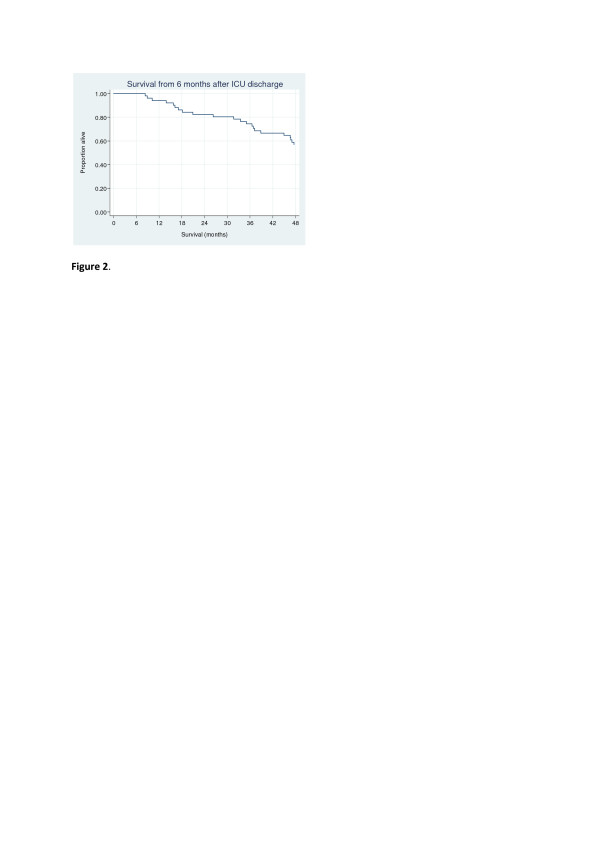
**Long-term survival in the chronic obstructive pulmonary disease ICU group**. Information on vital status was available for all patients.

The patient characteristics were split into those who were followed throughout the study and those who were lost to follow-up (Table 1). Twenty patients (39%) did not complete the study: nine died and eleven did not respond to questionnaires at 12 or 24 months (see study outline in Figure 1). Survival, which was followed for two years longer than HRQL, was roughly 40% at 48 months (Figure 2).

**Table 1:** Characteristics of the chronic pulmonary obstructive disease cohort^a^

Characteristics	Complete follow-up (*N* = 31)	Dead or lost to complete follow-up, (*N* = 20)
Males/females	12/19	6/14
Mean age, years (± SD)	69.7 (8.7)	70.7 (9.0)
APACHE II score, mean (± SD)	19.5 (6.7)	21.4 (8.2)
Charlson comorbidity index, mean (± SD)	3.7 (2.0)	4.2 (1.9)
Number of patients with invasive ventilator treatment (%)	13 (42%)	8 (40%)
Days in ICU, median (IQR)	2.2 (1.3 to 4.6)	2.0 (1.8 to 9.4)
Days in hospital, median (IQR)	10 (5 to 15)	13 (6.5 to 21)

^a^APACHE II, Acute Physiology and Chronic Health Evaluation II; IQR, interquartile range.

### Health-related quality of life

A detailed table with HRQL data is presented in the Additional file.

The median EQ-5D scores at six months were 0.66 (interquartile range (IQR) = 0.26 to 0.76) in the COPD-ICU group and 0.73 (IQR = 0.49 to 0.80) in the COPD reference group (*P* = 0.08 for difference between groups). The median EQ-VAS was 50 (IQR = 35 to 65) and 55 (IQR = 50 to 70) for the COPD-ICU and COPD reference groups, respectively (*P* < 0.05 for difference). There were no significant differences in the SF-36 dimensions between the COPD-ICU and COPD reference groups, although the difference in PF approached statistical significance (*P* = 0.059). Patients who were lost to follow-up after six months had low HRQL scores at six months (Table 2). The PF and SF scores of those who died were significantly lower than patients with complete follow-up (Table 2). Scores for patients who died were generally lower in all dimensions compared to patients who failed to respond to the questionnaire.

**Table 2:** Results of health-related quality of life measurement at six months in patients who had complete follow-up at twelve and twenty-four months (*n* = 31) or were lost to follow-up (dead = 9, no reply = 11)^a^

Parameters	Complete follow-up (*n* = 31)	Lost to follow-up	
		Dead (*n* = 9)	No reply (*n* = 11)
EQ-5D	0.66 (0.62 to 0.80) 0.62	-0.02 (-0.17 to 0.74) 0.22	0.55 (0.26 to 0.73) 0.51
EQ-VAS	50 (40 to 60) 51.1	39 (5 to 70) 36	60 (43 to 73) 55
PF	45 (25 to 65) 45.5	18 (3 to 33)* 21	35 (10 to 45) 34
RP	25 (0 to 75) 37.5	0 (0 to 25) 18	0 (0 to 33) 21
BP	47 (22 to 100) 53.6	22 (10 to 52) 35	41 (22 to 52) 46
GH	40 (25 to 52) 43.1	23 (10 to 40) 31	44 (30 to 67) 49
VT	45 (35 to 55) 47.0	30 (15 to 48) 34	50 (35 to 60) 50
SF	75 (38 to 100) 68.2	38 (13-63)* 38	50 (38 to 88) 57
RE	83 (0 to 100) 58.3	0 (0 to 42) 23	83 (0 to 100) 60
MH	68 (48 to 88) 66.0	58 (24 to 90) 56	72 (40 to 80) 60

^a^BP, bodily pain; EQ-5D, EuroQoL 5 Dimension; EQ-VAS, EuroQoL Visual Analogue Scale; GH, general health; MH, mental health; PF, physical functioning ; RE, role emotional; RP, role physical; SF, social functioning; VT, vitality. Values are medians (interquartile ranges) and means. *P < 0.05 compared to patients with complete follow-up.

**Figure 3:**
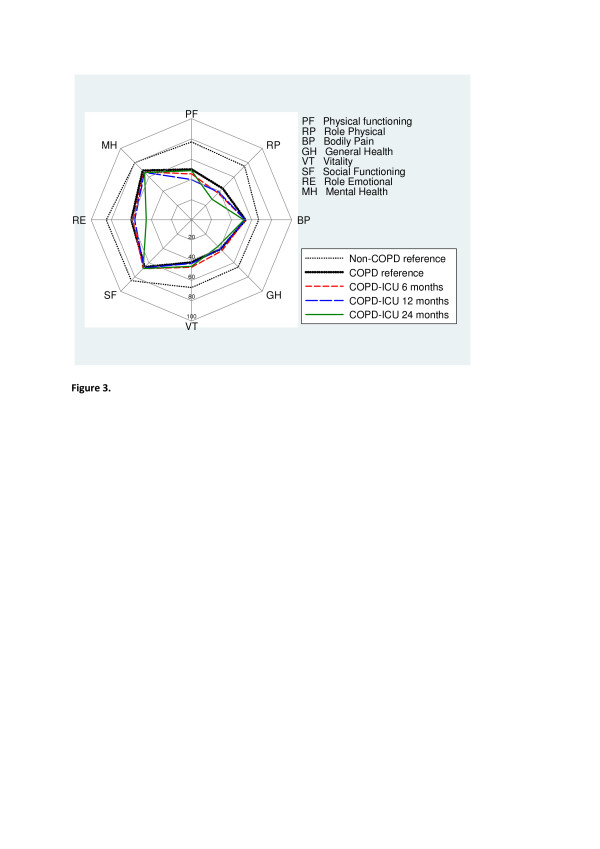
**Short Form 36 Health Survey measurements at 6, 12 and 24 months of patients in the chronic obstructive pulmonary disease ICU group providing responses at all three time points**. There were no statistically significant changes over time (analyzed using Friedman's test). The non-chronic obstructive pulmonary disease (non-COPD) and COPD reference groups are added for comparison. Values are means.

In patients with complete follow-up changes in HRQL score over time were minor. The median EQ-5D score was 0.66 at six months and remained unchanged at 12 and 24 months, whereas EQ-VAS scores were 51, 52 and 49 at 6, 12 and 24 months, respectively. Changes over time in the SF-36 dimensions are shown in Figure 3. Although always markedly lower than the non-COPD reference group, SF-36 dimensions were not significantly different from the COPD reference group with complete longitudinal follow-up (Figure 3).

## Discussion

There were three important findings in this prospective, longitudinal, multicentre study. First, measures of HRQL in COPD patients remained low but stable from six to twenty-four months after discharge from the ICU. Thus, we could not confirm our hypothesis of a decline in HRQL in this cohort of chronically ill patients. Second, HRQL in the COPD-ICU patient cohort was not significantly different from patients with COPD who had been hospitalized but not treated in ICU, that is, the COPD reference group. Third, HRQL among ICU patients who responded at six months but subsequently died was poor.

### Methodological issues

Before considering these findings, we need to discuss some methodological issues. The optimal way of securing a COPD diagnosis would have been spirometry prior to admission [18]. However, spirometry is often lacking in critically ill COPD patients. Previous studies have reported absence of spirometry in about 50% of patients deemed to have an acute exacerbation of COPD [19,20]. In the absence of spirometry, the diagnosis of COPD is typically based on the presence of clinical signs [19]. We identified 51 COPD patients in a cohort of 980 patients (5.2%) treated for more than 24 hours in the ICU. The higher-than-expected proportion of COPD patients [3] was probably due to selection of patients with an ICU stay longer than 24 hours in the basic study cohort.

Roughly 40% of the COPD cohort who responded six months after ICU discharge were dead at forty-eight months. This finding supports the notion that ICU patients with COPD have a poor long-term prognosis, given that the mortality rate during the first six months after discharge was similar to that reported in a prior Swedish study (46%) [3]. The subgroup of patients who died during the follow-up period had poor HRQL, stressing the need to analyze survivors and nonsurvivors separately when the evolution of HRQL over time is examined.

There are a number of disease-specific instruments for measuring HRQL and functional status in COPD [21]. The CAOS study and the Spanish study by Rivéra-Fernández *et al*. [2,5] employed such instruments. We chose to examine HRQL using generic instruments that are regularly employed in patients following intensive care [10]. The validity of the SF-36 has been shown repeatedly. SF-36 scores have been shown to reflect spirometric staging of disease [7,22], the St George's Respiratory Questionnaire [7,23] and at least two dyspnea scales [23,24]. Furthermore, the test-retest reliability of SF-36 in the intensive care population has been shown to be good [25,26]. We also used the EQ-5D, which has been recommended for evaluating ICU populations [10,15,27], to facilitate comparison with one of the few studies in this area: the UK CAOS study [5].

Analysis and reporting of HRQL is challenging. The EQ-5D and SF-36 measures provide composite scores derived from complex calculations. The eight separate SF-36 dimensions are based on 36 statements that yield data with wide distributions. The large number of statements typically lead to varying response frequencies, which is partly accounted for by the calculation algorithms. Parametric statistical methods are commonly employed despite ordinal measurements. This practice may lead to spurious findings [28]. Important and clinically significant differences may be missed when data are reported conventionally [29].

There is no consensus regarding when a difference in an SF-36 domain represents a clinically important difference. Dowdy *et al*. [30] referred to a difference of five points, but they also commented that a five-point difference could have different implications for different dimensions. Wyrwich *et al*. [31] suggested that a difference ranging from three to twelve points, varying with dimension, is clinically important in COPD patients.

SF-36 scores in the current study were occasionally low and had wide ranges, indicating significant floor and ceiling effects for some dimensions [17]. This is a known problem in the first version of SF-36, which we used, and is partly due to a limited number of questions and answers generating coarse scales in these dimensions. The wide distribution of data limits the possibility of identifying statistically significant patterns when the number of observations is limited, as is true of the current study.

### Health-related quality of life in chronic obstructive pulmonary disease patients after ICU care

Responses given six months after discharge from the ICU showed that patients lost to follow-up had lower HRQL scores, as indicated by their generally lower scores compared to those who completed the follow-up. These observations were similar whether assessed using the EQ-5D or the SF-36 (Table 2). Patients who died during the follow-up period had low HRQL scores compared to survivors with complete longitudinal follow-up. This difference was most obvious for the PF and SF dimensions, which is in line with prior research. A decline in PF was reported in patients with COPD, with a further decline in combination with higher comorbidity [32]. In a mixed Dutch ICU population, some aspects of HRQL recovered over six months, but the decreases in PF and SF remained [33].

In patients with a complete longitudinal follow-up, we found that HRQL was fairly stable from six to twenty-four months after intensive care. Moreover, it was quite similar to the COPD reference group who had not been treated in the ICU.

There are few studies on the development of HRQL over time in COPD patients after ICU care. In the CAOS study, Wildman *et al*. [5] reported slightly greater values for EQ-VAS at six months after ICU discharge compared to our study. Rivéra-Fernández *et al*. studied AECOPD patients after intensive care [2]. Mortality after discharge from the ICU was high, but they were able to find 50 patients (16.2% of the initial cohort) six years after intensive care. They found a decline in HRQL scores, but these scores were not significantly different from preadmission scores. Comparisons with our results, however, are difficult because Rivéra-Fernández *et al*. used an instrument developed within their research group.

HRQL in COPD patients is complex and dependent on several factors, such as dyspnea, depression, anxiety and exercise tolerance [21,34]. COPD reduces HRQL and more so with progression of the disease [7]. Patients with COPD and comorbidity experience further reductions in HRQL, particularly in the physical dimensions [32]. The COPD-ICU group in our study had a fairly high CCI comorbidity score [13], indicating that they had significant comorbid disease in addition to COPD. In ICU patients, factors often assumed to be important for HRQL after recovery, such as age, prolonged mechanical ventilation and ICU LOS, have not been shown to be of primary importance [35]. HRQL prior to ICU treatment and preexisting disease have both been found to be more relevant [12,30]. Surprisingly, in studies of COPD patients who had long-term home ventilation, blood gases and lung function were not shown to be related to HRQL [36,37].

An important strength of the present study is that we were able to follow HRQL at three time points. We also compared the results regarding HRQL in two reference groups: (1) an age- and sex-adjusted reference population (non-COPD reference) and (2) a COPD reference group from among the general patient population. A further strength is that we had information on comorbidity and illness severity at the time of ICU admission. Prior studies have stressed the importance of comorbidity for HRQL in COPD and ICU patients [12,32]. A weakness of this study, common to much work in this area, is the lack of information regarding premorbid HRQL and stage of COPD.

## Conclusions

Awareness of HRQL after critical illness is important if we are to avoid the notion that ICU treatment of COPD patients is futile and a burden for the patient and to society [38,39]. HRQL is difficult to forecast in critically ill COPD patients who survive ICU care, because information that is typically available, such as age, sex, ICU LOS, severity of illness and lung function parameters, is of limited value for predicting HRQL after recovery [30,35-37].

The present study showed that HRQL in COPD survivors after intensive care was lower than that in the general population but quite stable, and that it did not decline from six to twenty-four months after discharge from the ICU. HRQL at 24 months was similar to that of patients with COPD who had not received ICU treatment.

## Key messages

• HRQL in COPD patients treated in the ICU was low but did not decline further from six to twenty-four months after ICU discharge.

• HRQL in COPD patients 24 months after discharge from ICU was quite similar to that of COPD patients not treated in the ICU.

## Abbreviations

AECOPD: acute exacerbation of chronic obstructive pulmonary disease; APACHE II: Acute Physiology and Chronic Health Evaluation II; AQ20: 20-item Airways Questionnaire; BP: bodily pain; CAOS: COPD and Asthma Outcome Study; CCI: Charlson comorbidity index; COPD: chronic obstructive pulmonary disease; EQ-5D: EuroQoL 5 Dimensions; EQ-VAS: EuroQoL Visual Analogue Scale; GH: general health; HRQL: health-related quality of life; ICD-10: International Classification of Diseases, Tenth Revision; LOS: length of stay; MH: mental health; PF: physical functioning; RE: role emotional; RP: role physical; SF-36: Short Form 36 Health Survey; SF: social functioning; VAS: Visual Analogue Scale; VT: vitality.

## Competing interests

PN received travel expenses from Maquet and lecture fees from Orion Pharma.

## Authors' contributions

JB and SW designed the study, carried out statistical analysis, interpreted the results and drafted the manuscript. LE contributed to data analysis, interpretation of results and critical revision of the manuscript. LO, PN and FS participated in study design and contributed to data collection and critical revision of the manuscript. MF carried out statistical analysis and interpretation of the results. All authors read and approved the final manuscript.

## Supplementary Material

Additional file **The additional file table displays HRQL measurements from all patients that provided responses at 6, 12 and 24 months after ICU discharge**. Click here for file
