# Supplementary material for: A prospective longitudinal multicentre study of health related quality of life in ICU survivors with COPD
Source: Crit Care. 2013 Sep 24;17(5):R211. doi: 10.1186/cc13019 (PMC4056744; doi:10.1186/cc13019)
Supplement: Additional file — The additional file table displays HRQL measurements from all patients that provided responses at 6, 12 and 24 months after ICU discharge. [file cc13019-S1.doc]

**Additional file 1: Table.**

Results of HRQL measurements at 6, 12 and 24 months after ICU discharge. Data from all patients providing responses are shown. Values are median (IQR), mean and number of observations. There were no statistically significant differences between measurements at 6, 12 and 24 months (Kruskal-Wallis’ test).

|  | COPD-ICU group | | | General population | |
| --- | --- | --- | --- | --- | --- |
|  | 6 months after discharge  N=51 | 12 months after discharge  N=37 | 24 months after discharge  N=31 | COPD reference  N=38 | Non-COPD reference  N=51 |
| EQ-5D | 0.66  (0.26-0.76)  0.52 | 0.66  (0.33-0,73)  0.55 | 0.66  (0.38-0.73)  0.55 | 0.73  (0.49-0.80)  0.61 | 0.79  (0.77-0.81)  0.79 |
| EQ-VAS | 50  (35-65)  48.1 | 50  (38-65)  51.3 | 50  (40-60)  50.7 | 55  (50-70)  57.7 | 75.0  (74.1-75.8)  75.1 |
| PF | 35 (15-50)  35.8 | 40 (15-60)  39.6 | 55 (30-70)  50.7 | 50 (20-80)  49.8 | 76 (73-79)  76.4 |
| RP | 0 (0-50)  24.6 | 25 (0-50)  30.3 | 0 (0-75)  30.4 | 12.5 (0-100)  43.8 | 75 (72-76)  74.6 |
| BP | 41 (22-88)  49.1 | 41 (28-84)  51.4 | 46 (27-74)  52.0 | 51 (41-73)  54.2 | 67 (65-69)  67.2 |
| GH | 35 (20-56)  41.2 | 40 (25-60)  42.0 | 35 (18-50)  37.5 | 42 (30-50)  40.8 | 66 (64-67)  65.7 |
| VT | 45 (33-55)  45.1 | 40 (30-55)  44.1 | 48 (28-68)  46.5 | 45 (26.7-60)  42.4 | 65 (65-70)  66.7 |
| SF | 63 (38-88)  58.2 | 75 (44-88)  64.6 | 75 (50-94)  67.2 | 68.8 (50-87.5)  65.6 | 84 (84-87)  84.9 |
| RE | 33 (0-100)  48.5 | 50 (0-100)  53.1 | 33 (0-100)  47.0 | 100 (0-100)  59.8 | 84 (83-87)  84.8 |
| MH | 64 (44-82)  62.3 | 70 (48-88)  64.9 | 70 (44-86)  63.5 | 72 (56-84)  68.5 | 79 (79-82)  79.9 |
